# Sclareolide as Antifungal Strategy Against *Cryptococcus neoformans*: Unveiling Its Mechanisms of Action

**DOI:** 10.3390/microorganisms12112324

**Published:** 2024-11-15

**Authors:** Arumugam Ganeshkumar, Patricia Michelle Nagai de Lima, Jebiti Haribabu, Bruno Montanari Borges, Nycolas Willian Preite, Flavio Vieira Loures, Arunachalam Arulraj, Juliana Campos Junqueira

**Affiliations:** 1 Department of Biosciences and Oral Diagnosis, Institute of Science and Technology, Sao Paulo State University (UNESP), Sao Jose dos Campos, São Paulo 12245-000, Brazil; patricia.nagai@unesp.br; 2 Department of Materials Physics, Saveetha School of Engineering, Saveetha Institute of Medical and Technical Sciences (SIMTS), Thandalam, Chennai 602105, Tamil Nadu, India; 3 Faculty of Medicine, University of Atacama, Los Carreras 1579, Copiapo 1532502, Chile; 4 Chennai Institute of Technology (CIT), Chennai 600069, Tamil Nadu, India; 5 Institute of Science and Technology, Federal University of Sao Paulo (UNIFESP), Sao Jose dos Campos, Sao Paulo 12231-280, Brazilpreite@unifesp.br (N.W.P.);; 6 Departamento de Electricidad, Facultad de Ingeniería, Universidad Tecnológica Metropolitana (UTEM), Av. José Pedro Alessandri 1242, Ñuñoa, Santiago 7800002, Chile; arul@utem.cl

**Keywords:** *Cryptococcus*, phytochemicals, sclareolide, antifungals, ROS and MMP

## Abstract

Cryptococcal infection commonly begins as an opportunistic infection in humans, however, this can escalate to a systemic or life-threatening form in immunocompromised individuals. Here, we aim to identify novel antifungal molecules from plants resources. Sclareolide, a phytochemical classified as a sesquiterpene lactone, was assessed against *Cryptococcus neoformans* H99. Sclareolide exhibited promising antifungal properties with a minimum inhibitory concentration (MIC) of 16 µg/mL. Additionally, the *C. neoformans* growth rate was significantly affected by sclareolide treatment in a concentration-dependent manner, as observed through a time killing assay, with a significant reduction at MIC × 8 compared to the control by 48 h. To elucidate the underlying mechanisms of sclareolide antifungal activity, fluorescence-based methods were employed. Propidium iodide (PI) accumulation assay indicated a reduction in C. *neoformans* membrane integrity, with values as low as 6.62 ± 0.18% after treatment. Moreover, sclareolide at MIC × 4 and MIC × 8 significantly increased the production of reactive oxygen species (ROS) and reduced the mitochondrial membrane potential (MMP), suggesting oxidative stress and mitochondrial dysfunction in *C. neoformans*. Sclareolide did not induce caspase-dependent apoptosis, suggesting a non-apoptotic mechanism. Further, a checkerboard experiment was performed to assess potential synergistic interaction with Amphotericin B, however, no synergism was observed. Moving on, sclareolide at 128 µg/mL did not exhibit toxicity in *Galleria mellonella,* further supporting its potential as a safe antifungal agent. These findings suggest that the antifungal activity of sclareolide against *C. neoformans* is mediated by oxidative stress. Further in vivo and pharmacokinetic studies are recommended to explore the potential of sclareolide as a prototype for the development of novel anti-cryptococcal therapies.

## 1. Introduction

Cryptococcosis is a fungal infection primarily caused by *Cryptococcus neoformans* and *Cryptococcus gattii*, which is an encapsulated fungus found in the environment [1]. Unlike other fungal pathogens, *Cryptococcus* spp. are typically sourced from the soil, pigeon droppings, and decaying wood [2] and have been recognized as the most predominant opportunistic fungus, causing significant morbidity and mortality in humans. Amongst them, *C. neoformans* is considered an important fungal pathogen according to the World Health Organization (WHO) fungal pathogen priority list. A key factor in the virulence of *C. neoformans* is its polysaccharide capsule, and therefore is considered a crucial factor in anti-fungal drug discovery. This capsule plays a pivotal role in enabling fungal cells to evade the host immune system. Additionally, it modulates the host’s immune response and provides protection against environmental stress, making it a key target for therapeutic intervention [3,4]. Cryptococcosis begins with the inhalation of cryptococcal spores, potentially leading to life-threatening pulmonary or neurological infections [5]. In healthy individuals, the cryptococcal spores may reside in the lungs without harming the host, however, in immunocompromised individuals, specifically HIV-positive patients, it affects the lungs and nervous system [6,7]. Globally, cryptococcal meningitis alone accounts for an estimated 223,100 cases and 181,100 deaths annually [8] with untreated cryptococcosis having a 100% mortality rate [9]. The treatment regimen for invasive cryptococcosis depends solely on three antifungal agents, which can be administered individually or in combination. Amphotericin B with flucytosine is the most frequently used combination for neuro-cryptococcosis prophylaxis [5,10].

However, the treatments of fungal infections are threatened by the emergence of antifungal resistance [11]. The growing prevalence of drug-resistant fungal infections has spurred an urgent need for new antifungal therapeutic options. Rather than focusing exclusively on the development of novel antifungal agents, current research is exploring the potential of existing molecules as alternative antifungal therapies. In this context, natural agents are found to be the promising candidates for the development of cost-effective antifungal products [12]. Natural agents derived from various flora, fauna, and some microorganisms offer a promising alternative to synthetic antifungals by showcasing superior activity from antibacterial to anticancer, representing a rich source of bioactive scaffolds [13]. Currently, phytochemicals are being explored because of their unique biological and chemical properties, which yield several medically important bioactive compounds, such as morphine, artemisinin, caffeine, and essential oils from aromatic plants [14,15,16].

Numerous investigations have focused on elucidating the antifungal properties of crude plant extracts, serving as a foundation for developing plant-based antifungal therapies. Several plant species, including *Kigelia africana* [17], *Terminalia mantaly* [18], *Eriobotrya japonica* [19], *Tulbaghia violacea* Harv [20], *Annona coriacea* Mart [21], *Eremophila alternifolia* [22] and *Pelargonium sidoides* [23] have been identified as a potential source of anticryptococcal compounds. Additionally, Brunei Propolis [24], plant essential oils [25,26,27] and their bioactive principles like eugenol, isoeugenol [28], and carvacrol [29] have shown promising antifungal properties against *C. neoformans* [30,31,32].

Medicinal plants and plant-based products have been valued for their economic importance for the past few decades [33,34,35]. However, crude or purified phytochemicals can be toxic to the host. Therefore, toxicity testing is a crucial step before further utilization. Given this, various host models have been developed to assess the toxicity of chemical compounds, but the use of mammals, such as mice and rabbits, in toxicological research is often expensive and labor-intensive [36,37]. Recently, the invertebrate *Galleria mellonella*, the Greater Wax Moth, has emerged in clinical and toxicological research due to its unique pathophysiology [38,39,40]. Compared to other host models, *G. mellonella* offers several advantages, such as cost -effectiveness, ease of use, reduced ethical concerns and an innate immune response similar to that of mammals, making it a more practical option for research [41].

**Figure 1:**
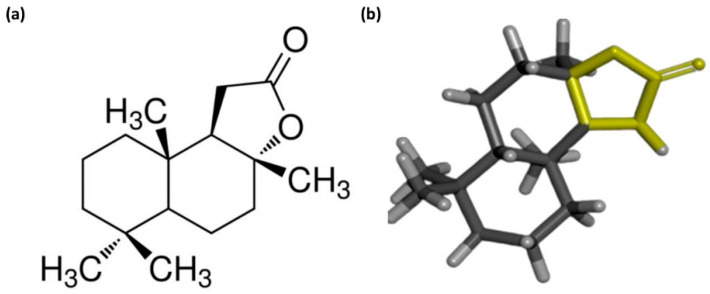
The 2D chemical structure of sclareolide, including its potential functional groups responsible for antifungal mechanisms, is illustrated in (**a**). In the 3D chemical structure, the lactone group is highlighted in yellow and the decalin ring in grey (**b**).

This study aimed to identify a novel compound with antifungal activity against *C. neoformans* that exhibits no toxicity to animal cells, specifically investigating sclareolide, which is a sesquiterpene lactone naturally isolated from various plant sources. The chemical structure of sclareolide consists of different functional groups (Figure 1a,b). Importantly, lactone and decalin are the prime contributors to its antifungal properties. Lactones are groups of organic compounds with wide ranges of biological properties, including antimicrobial [42,43], anti-inflammatory [44], and anticancer [45]. Additionally, we explored the mode of action of sclareolide using fluorescence spectrometer and flow cytometry-based assays. Beyond evaluating the antifungal effects of sclareolide, we also assessed its toxicity through *G. mellonella* larvae, providing comprehensive insights into its antifungal efficacy and safety.

## 2. Materials and Methods

### 2.1. Strains, Media, Chemicals, and Kits

*C. neoformans* H99 was the strain used in all in vitro assays; however, *Cryptococcus gattii* R265 was also included in the minimum inhibitory concentration (MIC) assay. Sabouraud dextrose agar (SDA) and 96-well cell culture plates were sourced from Kasvi, Pinhais, Brazil. Dimethyl sulfoxide (DMSO), sclareolide, rhodamine 123 (Rho 123), 2′,7′-dichlorofluorescin diacetate, propidium iodide (PI), Roswell Park Memorial Institute medium (RPMI-1640), Amphotericin B and Triton X-100 were purchased from Sigma Aldrich (St. Louis, MO, USA), Merck (Rahway, NJ, USA). Additionally, we used the Dead Cell Apoptosis Kits with Annexin V for Flow Cytometry (Catalog number V13242, Invitrogen, Waltham, MA, USA) and CaspACE™ FITC-VAD-FMK In Situ Marker (Catalog number G7461, Promega, Madison, WI, USA).

### 2.2. MIC Determination

The Clinical and Laboratory Standards Institute (CLSI) method was used to determine the MIC of the test compounds [46,47]. Briefly, the fungal strains of *C. neoformans* and *C. gattii* were recovered from frozen stock solutions and cultured on SDA plates containing 50 µg/mL of chloramphenicol for 48 hrs. Then, cells were washed three times using sterile saline (0.9% NaCl), and cell density was adjusted to 4 × 10^4^ cells/mL. Stock solution of the test compounds were prepared in DMSO. Next, the compounds were serially diluted in a 96-well plate using RPMI medium, and the final concentrations in the wells ranged from 1024 µg/mL to 1 µg/mL for sclareolide and 100 µg/mL to 0.195 µg/mL for Amp B. Subsequently, 100 µL of the cell suspension was added to each well, except in the sterile control. The MIC was defined as the lowest concentration of sclareolide that visually inhibited the viable growth of the test strains compared to the growth control after 48 h of incubation at 37 °C.

### 2.3. Time Killing Kinetics

Following the protocol by Levorato-Vinche et al. [48], the growth profile of *C. neoformans* H99 was assessed after treatment with sclareolide. Briefly, in a 24-well plate, *C. neoformans* was incubated in RPMI medium and was treated with sclareolide at MIC, MIC × 4, and MIC × 8. Wells without any test compound served as growth controls, while wells with only RPMI medium served as sterility controls. At 0, 2, 4, 6, 8, 24, and 48 h of incubation, 10 µL of sample was collected from each well and plated in SDA plates. For the 8, 24, and 48 h time points, due to a significant increase in cell numbers, samples were serially diluted in sterile saline before plating on SDA plates. After 48 h of incubation, the viability were assessed by colony forming units (CFU) and the results were plotted [49].

### 2.4. Determination of ROS and MMP by Fluorescence Analysis

The levels of reactive oxygen species (ROS) and mitochondrial membrane potential (MMP) in *C. neoformans* H99 cells treated with sclareolide were assessed using ROS and MMP-selective fluorescent probes [50,51]. Briefly, 1 × 10^6^ cells/mL of fungal cells were mixed with 16, 32, 64, and 128 µg/mL of sclareolide in the RPMI medium. The plates were incubated at 37 °C for 24 h. After incubation, 10 µM of 2′,7′-dichlorofluorescin diacetate was added to each well to detect ROS production and incubated for 30 min at room temperature in dark. The fluorescence was measured at 485 nm (excitation) and 530 nm (emission) [50]. Similarly, to assess MMP, 5 µg/mL of Rho 123 was added in each well and incubated for 30 min at room temperature in dark. The fluorescence was measured using Varioskan™ LUX multimode microplate reader (Thermo Scientific, Waltham, MA, USA) at 511 and 534 nm for excitation and emission [51].

### 2.5. Study of Membrane Integrity, Apoptosis, and Caspase Activity Using Flow Cytometer Assays

A concentration of 1 × 10^6^ cells/mL fungal cells were treated for 24 h with sclareolide at 16, 32, 64, or 128 µg/mL or with Amp B at 0.25 µg/mL. After treatment, cells were washed thrice with sterile saline and collected by centrifugation at 5000 rpm for 10 min. The same treatment condition and procedure were followed to prepare cells to analyze membrane integrity, apoptosis, and caspase activity.

For the membrane integrity analysis, the resulting pellet was mixed in 500 µL saline containing 1 µg/mL of PI. Following a 30 min incubation, the cells were analyzed using a BD FACS Lyric flow cytometer. Heat-killed cells (prepared by incubating cells at 80 °C for 20 min) were used to distinguish between PI-positive (dead) and PI-negative (live) cells [47].

For the apoptosis assay, the treated cells were washed thrice with sterile saline and mixed with lyticase enzyme at least 300 IU/mL (L2524, Sigma-Aldrich, Burlington, MA, USA) and incubated at room temperature for one hour. Further, the cells were washed and resuspended in 100 µL 1 × annexin binding buffer. The staining was performed following the protocol provided by the manufacturer (FITC Annexin V/Dead Cell Apoptosis Kit, Invitrogen, USA). Briefly, the cell suspension was mixed with 25 µL of FITC Annexin V and 5 µL of PI and incubated in the dark for 15 min. Prior to sample acquisition, the cell suspension was further diluted to 500 µL using 1 × annexin binding buffer [52].

To assess caspase activity, cell pellets were prepared as described above, stained with 10 µM CaspACE™ FITC-VAD-FMK In Situ Marker (Promega Corporation, USA), prepared and analyzed according to the protocol described by the manufacture [52].

For all three assays, at least 50,000 events were analyzed in medium scanning mode using a flow cytometer (BD FACS Lyric ™ Becton, Dickinson and Company, Franklin Lakes, NJ, USA).

### 2.6. Checkerboard Synergy Assay

The synergistic activity between sclareolide and Amp B was evaluated using a checkerboard microtiter assay, following the method described by Jafri and Ahmad [53]. Briefly, stock solutions of sclareolide and Amp B were first prepared in DMSO, and then diluted in RPMI medium to achieve working solutions at four times their respective MICs. In a 96-well plate, 50 µL of Amp B was added to wells A1 to H1. In another 96-well plate, 50 µL of sclareolide was added to wells A1 to A10 and serially diluted using RPMI medium. Each well was then filled with appropriate concentrations of both sclareolide and Amp B, along with *C. neoformans* H99 cells at a concentration of 2 × 10^4^ cells/mL. The plates were incubated at 37 °C for 24–48 h. After incubation, 5 µL from each well was plated on SDA plates, and viable growth was observed after 48 h. Wells without colony growth were considered to indicate no viable cells. The interaction between sclareolide and Amp B was assessed based on the fractional inhibitory concentration index (FICI). Interactions were classified as synergistic (FICI ≤ 0.5), additive (0.5 < FICI < 1), indifferent (1 ≤ FICI < 4), or antagonistic (FICI ≥ 4), as described by Lewis et al. [54].

### 2.7. In Vivo Toxicity in Galleria Mellonella Model

*G. mellonella* larvae, weighing between 200 and –250 mg, were obtained from the Invertebrate Laboratory at ICT-UNESP, São José dos Campos, and were free of any signs of infection or abnormalities [55]. The larvae were separated into groups of ten. Four different concentrations of sclareolide and Triton X-100 were prepared in sterile saline. Prior to injection, the larvae’s injection site (left proleg) was cleaned with 70% ethanol. Each larva was injected with 10 µL of the test compound. The larvae were monitored for five days, and mortality rates were recorded to generate survival curves. The group of larvae that received 10 µL of saline served as the negative control, while those injected with 0.5% to 5% Triton X-100 were designated as the positive control for survival rate.

### 2.8. Statistical Analysis

All experiments were performed independently at least three times to ensure statistical significance. One-way ANOVA was used to compare the statistical significance among the test groups, which was followed by Tukey’s multiple comparison test to identify significant differences between groups. Kaplan–Meier survival analysis was employed to generate survival curves for the in vivo toxicity assays. All the statistical analyses were conducted using GraphPad Prism 9.5.1.

## 3. Results

### 3.1. Sclareolide Has Antifungal Activity Against C. neoformans with an Extended Spectrum for C. gattii 

In the susceptibility assay, *C. neoformans* H99 was more susceptible to sclareolide with an MIC of 16 µg/mL whereas *C. gattii* R265 demonstrated a higher MIC of 32 µg/mL. In contrast, Amp B demonstrated superior activity against both *C. neoformans* H99 and *C. gattii* R265, with MIC of 0.25 µg/mL.

### 3.2. Sclareolide Inhibits the Growth of C. neoformans

**Figure 2:**
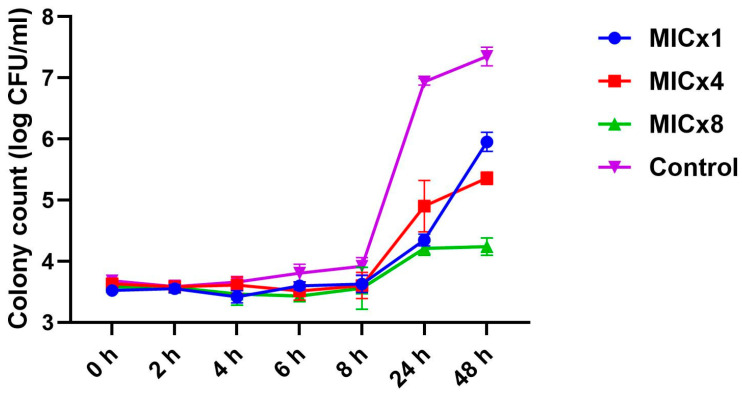
A time-kill curve for *Cryptococcus neoformans* H99 was generated after exposure to 16, 64, and 128 µg/mL of sclareolide. A 10 µL aliquot from each treatment was plated on SDA plates to determine CFU counts. Treatment with sclareolide led to a significant, concentration-dependent reduction in CFUs of *C. neoformans* H99.

Time-kill kinetics were performed to understand the impact of sclareolide on *C. neoformans* H99. The variations in the growth profile of *C. neoformans* H99 cells exposed to different concentrations of sclareolide, as well as the control, are shown in Figure 2. The growth rate of *C. neoformans* H99 remained constant across all groups during the first two hours of treatment. By the 4th hour, the control group showed a consistent increase in growth, which continued until the 48th hour. In contrast, at the 4th hour of treatment, sclareolide significantly inhibited the growth of *C. neoformans* H99 in all tested concentrations (MIC × 1, MIC × 4 and MIC × 8). Since the CFU of *C. neoformans* H99 did not reach zero after 48 h of exposure to sclareolide, it is considered fungistatic in nature.

### 3.3. Sclareolide Exposure Reduces the Membrane Permeability and Increases ROS Production in C. neoformans H99

**Figure 3:**
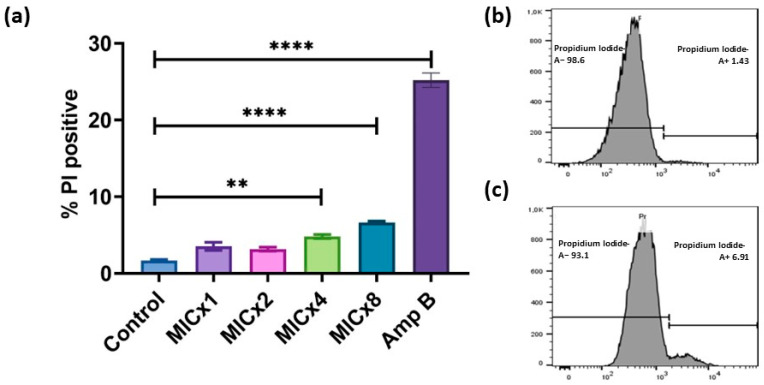
Effect of sclareolide and amphotericin B on the membrane integrity of *C. neoformans* H99. (**a**) No significant loss of membrane integrity was observed in cells treated with sclareolide at 16 or 32 µg/mL. However, a gradual increase in membrane damage was observed at 64 µg/mL (** *p* = 0.004) and 128 µg/mL (**** *p* < 0.0001), which was statistically significant compared to the control group at a 99.99% confidence level. PI staining was performed for (**b**) the control group and (**c**) the group treated with 128 µg/mL of sclareolide.

The possible antifungal mechanisms of sclareolide against *C. neoformans* H99 were evaluated using a flow cytometer through membrane permeable fluorescent probes. The cell membrane of yeast serves as the primary defense against environmental stimuli. PI is impermeable to cells with an intact membrane; however, when their cell membrane is damaged, PI can enter into these yeast cells, indicating cellular damage. After treatment with different concentrations of sclareolide and Amp B, the level of red fluorescence emitted by PI was analyzed and is presented in Figure 3. As shown in Figure 3, *C. neoformans* H99 treated with sclareolide at 16–128 µg/mL compared to control group, the percentage of PI-positive cells gradually increased from 3.55 ± 0.53% to 6.62 ± 0.18%, while treatment with Amp B at 0.25 µg/mL resulted in a 25.2 ± 0.9% increase in PI-positive cells, which is indicative of strong membrane damage.

**Figure 4:**
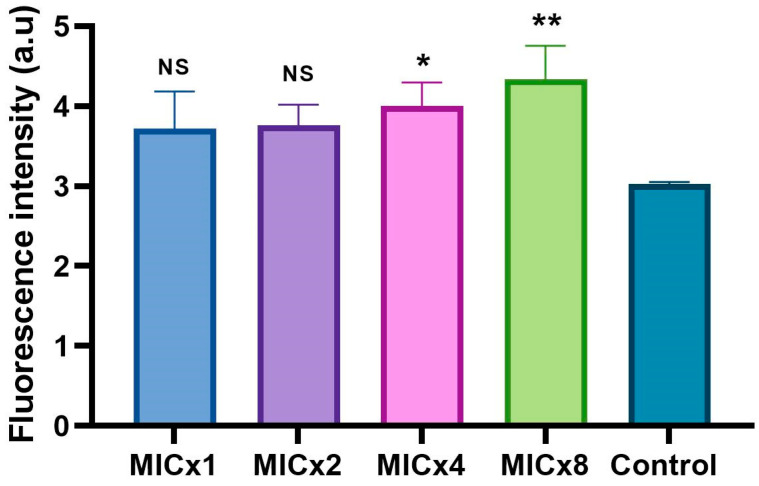
The level of reactive oxygen species (ROS) in *C. neoformans* H99 cells following exposure to different concentrations of sclareolide was assessed using 2,7-dichlorofluorescin diacetate. Similar to the PI staining results, no statistically significant increase in ROS was observed at 16 or 32 µg/mL of sclareolide. However, ROS generation was significantly elevated at 64 (*p* = 0.03) and 128 µg/mL (*p* = 0.004). NS-non significance; * = *p* < 0.05 and ** = *p* < 0.01.

Further, the induction of ROS and its association with cell death was studied. Fungicidal compounds typically generate elevated levels of ROS, leading to cell death, whereas this effect is less certain for fungistatic compounds. Variations in ROS production following treatment with sclareolide are shown in Figure 4, based on the levels of green fluorescence. Sclareolide, at MIC and MIC × 2 induced ROS production; however, these values were not significantly different from the growth control (*p* > 0.05). In contrast, a significant increase in ROS was observed when the concentration of sclareolide was raised to MIC × 4 (*p* = 0.03) and MIC × 8 (*p* = 0.0048).

### 3.4. Sclareolide Exposure Reduces the Mitochondrial Membrane Potential

**Figure 5:**
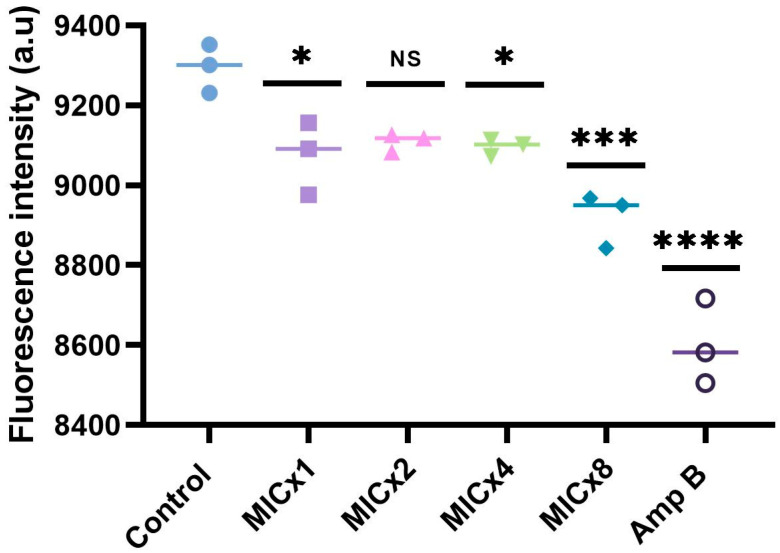
Assessment of mitochondrial membrane potential (MMP) in *C. neoformans* H99 cells was conducted using Rhodamine 123 staining. The graph illustrates the results after 24 h of incubation with varying concentrations of sclareolide and amphotericin B. Statistical significance is indicated as follows: * *p* < 0.05; *** *p* = 0.0005; **** *p* < 0.0001; NS—non significance.

Disturbance in MMP is a hallmark of cell death. Continuing from the previous experiment, the MMP of sclareolide exposed cells was quantified using Rhodamine 123, which is a cationic dye that selectively accumulates in the mitochondria of living cells due to the negative potential inside the mitochondria. The accumulation of Rho 123 is directly proportional to the MMP, leading to an increased green fluorescence in viable cells. Changes in the MMP in *C. neoformans* H99 cells treated with sclareolide, and Amp B are shown in Figure 5. Alterations in MMP were observed in *C. neoformans* H99 cells exposed to different concentrations of sclareolide (MIC, MIC × 4 and MIC × 8) and the Amp B group, compared to the control group. The depolarization of the mitochondrial membrane was evidenced by a reduced fluorescence rate upon treatment. However, these values were not enough to induce apoptosis mediated cell death, which may be related to the fungistatic mechanism of sclareolide.

### 3.5. Loss of Viability of C. neoformans H99 Is Not Associated with Apoptosis

**Figure 6:**
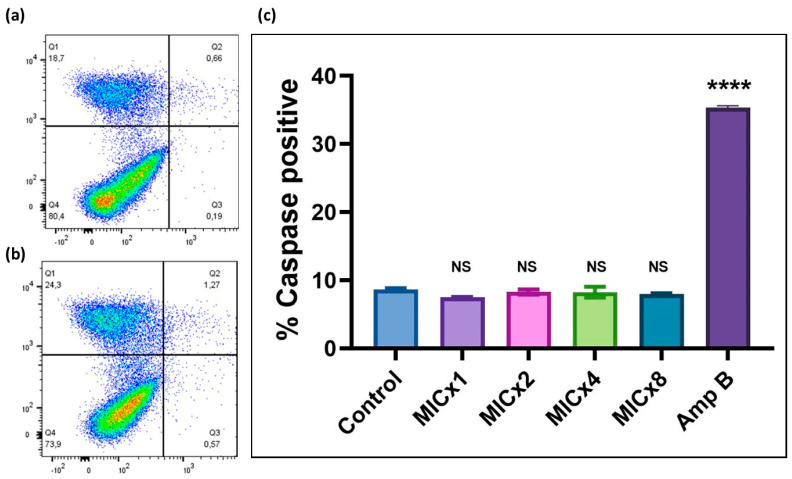
Phosphatidylserine externalization, an early marker of apoptosis, was assessed using FITC-annexin V and PI staining. Cells were categorized based on fluorescence signals as follows: annexin V+/PI+ (apoptotic), annexin V−/PI+ (necrotic), and annexin V−/PI− (viable). (**a**) Sclareolide-treated groups showed no significant increase in annexin V+/PI+ cells, indicating no apoptotic events. (**b**) FITC-annexin V and PI staining of the control. (**c**) The graph shows caspase-positive cells following treatment with the test compound, which was detected using the CaspACE™ FITC-VAD-FMK In Situ Marker. Since no apoptosis was observed, only an insignificant proportion of FITC-positive cells was detected. In contrast, amphotericin B treatment confirmed caspase-positive cells, indicating caspase-dependent apoptotic cell death (**** *p* < 0.0001). NS—non-significance.

To further confirm the fungistatic potential of sclareolide, we evaluated apoptotic-like events in *C. neoformans* H99. Cells treated with varying concentrations of sclareolide were assessed for the presence of the anionic phospholipid phosphatidylserine, which is a marker of apoptosis. In apoptotic cells, the disturbance of the plasma membrane leads to the translocation of phosphatidylserine. However, even at high concentration (128 µg/mL), sclareolide did not induce significant apoptosis, as indicated by Annexin V+/PI+ staining, compared to the untreated control group (Figure 6a,b).

Caspases are protease enzymes critical to the execution of apoptosis, therefore, to further investigate if sclareolide can induce apoptotic pathways, cells were stained with the pan-caspase inhibitor, CaspACE™ FITC-VAD-FMK, which fluoresces upon activation of caspases. In our study, sclareolide at concentrations ranging from 16 to 128 µg/mL did not induce caspase activity, confirming that sclareolide does not trigger caspase-dependent apoptotic cell death. In contrast, Amp B, a known apoptotic inducer in fungal cells, demonstrated a significant increase in caspase-positive cells (35.33 ± 0.29%), with statistical significance compared to the untreated control group (*p* < 0.0001) (Figure 6c).

### 3.6. Sclareolide Combined with Amp B Reduced Cell Viability of C. neoformans H99

To assess the synergistic interaction between sclareolide and Amp B, a checkerboard microdilution assay was performed. The previously determined MIC values of sclareolide and Amp B were 16 µg/mL and 0.25 µg/mL, respectively. When Amp B is combined with sclareolide, the MIC of Amp B was reduced by several folds, down to 0.0009 µg/mL. The efficacy of the combinations was assessed using the Fractional Inhibitory Concentration Index (FICI), which predicts the nature of their interaction as synergistic, additive, or antagonistic (as detailed in Section 2). All combinations tested in this study were classified as having an indifferent effect, as their FICI values fell around >1 (Table 1).

### 3.7. Sclareolide Is Non-Toxic to Galleria mellonella

**Figure 7:**
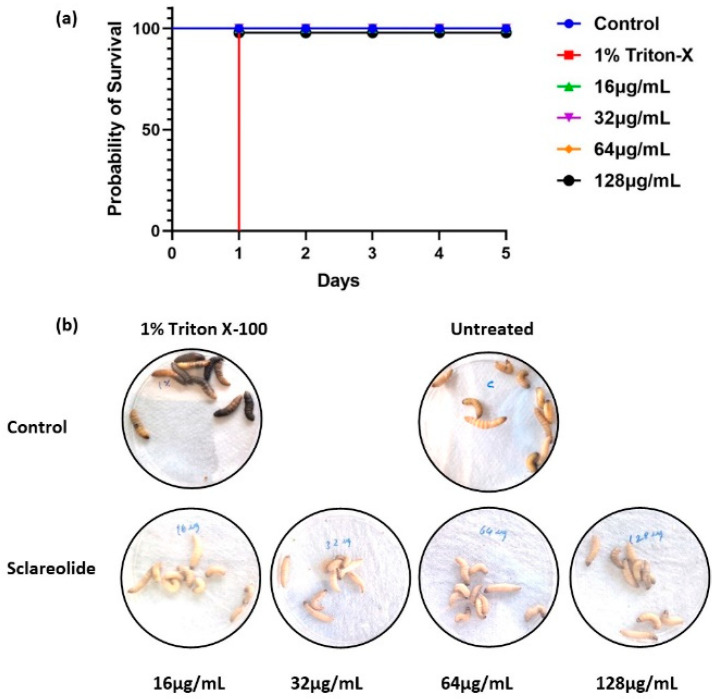
Toxicological assessment of sclareolide and Triton X-100 in *G. mellonella*. A toxicological study was conducted using *G. mellonella* larvae with each experimental group consisting of 10 larvae. The larvae were injected into varying concentrations of sclareolide and Triton X-100. Over a period of five days, the survival rate was monitored. Larvae exhibiting no movement and complete melanization were considered dead. (**a**) A Kaplan–Meier survival curve was generated to illustrate the survival proportion across the different treatment groups. (**b**) The phenotypic appearance of the larvae following injections with different concentrations of sclareolide, and Triton X-100 was recorded and presented to show the visual effects of toxicity.

The toxicity of sclareolide in *G. mellonella* larvae was assessed after injection with 10 µL of varying concentrations of sclareolide (MIC × 1, MIC × 2, MIC × 4, and MIC × 8) and Triton X-100 (0.5%, 1%, 3%, and 5%). The survival rate of the treated larvae was monitored over five days, with immobile larvae considered dead. The number of dead larvae in each group was recorded, and a Kaplan–Meier survival curve was generated. No deaths were observed in the groups treated with sclareolide up to MIC × 4, however, a slight reduction in survival was noted at MIC × 8, indicating the generally non-toxic nature of sclareolide (Figure 7a). In contrast, immediate death occurred in the groups treated with Triton X-100- at 3% and 5%, while the 1% group lost complete viability within 24 h (Figure 7a,b).

## 4. Discussion

Phytochemicals have been known for their biological properties since ancient times. Even now, significant efforts are being made to prove their effectiveness in different aspects. Recent studies have shown that phytochemicals like curcumin [56,57], quercetin [58], berberine [59], dioscin [60,61], ellagic acid [62,63], and essential oils from aromatic plants [25,26,27,53,64] have antifungal potential against most common fungal pathogens. Sclareolide, a sesquiterpene lactone, is primarily isolated from various plants, such as *Salvia sclarea*, and can also be bio-transformed from its derivatives [65,66,67,68]. It is known for diverse biological properties, including antiviral, antifungal, and anticancer activities [66,68,69]. Additionally, it is used in the cosmetics and food industries [70].

To better understand this, the antifungal properties of sclareolide observed in this study were compared with earlier findings on sesquiterpenoids and related compounds. Recent research has shown fungicidal activity of drimane sesquiterpenoid compounds synthesized from sclareolide against medically important fungus. Among these, drimenol exhibited fungicidal activity against *C. albicans*, *Cryptococcus*, and dermatophytes, with a MIC ranging from 4 to >64 µg/mL [30]. In the present study, sclareolide displayed a MIC of 16 µg/mL against *C. neoformans* H99, this is within the MIC ranges reported by drimenol. But drimenol was reported to be fungicidal, whereas sclareolide is fungistatic, indicating that substantial modification of sclareolide structure may enhance its antifungal potential. To support this argument, labdane-type diterpenes synthesized from sclareolide and sesquiterpenoids, such as polygodial and warburganal, were active against most common fungal pathogens [67,68]. This further supports the structural modification of sclareolide to increase its potential as well as antifungal spectrum.

In general, sesquiterpenes are fungicidal in nature. In this study, we are the first to describe the fungistatic effects of sclareolide on *C. neoformans* H99. Supporting this, polygodial, a drimane sesquiterpene aldehyde isolated from the bark of *Drimys winteri*, demonstrated fungistatic activity against *Candida* species isolated from patients with candidemia [71] Sclareolide exhibits promising fungistatic properties, as it effectively inhibits the growth of *C. neoformans* H99 cells without causing immediate cell death. This inhibition suggests that sclareolide interferes with essential cellular processes, aligning with its recognized fungistatic behavior. It is well-established that many phytochemicals and antifungals, such as Amp B, induce apoptosis in *Cryptococcus* species. However, even at higher concentrations, sclareolide does not cause immediate cell death but instead leads to a progressive loss of cell viability, as confirmed by an annexin-V apoptosis detection assay.

Further, the results of the present study demonstrate the fungistatic effect of sclareolide on *C. neoformans* H99 with minimal impact on membrane integrity, as evidenced by approximately 6.62% of PI positive cells at 128 µg/mL. This mild stress on *C. neoformans* cell membrane will likely contribute to the mechanism of action of sclareolide. Thus inhibiting the growth of fungi without causing cellular or biological events. These findings aligned with earlier studies, suggesting that the antifungal potential of sclareolide does not depend on the loss of membrane integrity [72]. In contrast, drimenol, a broad-spectrum antifungal agent derived from sclareolide, has demonstrated superior activity by disturbing the membrane integrity of fungi [30].

Isobavachalcone is a flavonoid compound primarily isolated from *Psoralea corylifolia*, as evidenced by several biological activities. At low concentrations, isobavachalcone increases the production of cellular ROS in *C. neoformans* H99 cells. The proportion of ROS positive cells is increased from 47.9% to 94.32% within 1–4 h of treatment [73]. In the same way, the cells exposed to sclareolide tested positive for increased ROS production in a concentration-dependent manner. At MIC, sclareolide induced 22.86 ± 14.87% ROS production, while ROS production reached up to 43.10 ± 12.78% in MIC × 8. These variations in ROS might be aligned with fungistatic behavior of sclareolide, since isobavachalcone is fungicidal to *C. neoformans* H99. Similar observations were reported by Spadari et al., who treated *C. neoformans* H99 cells with miltefosine, an organic compound originally developed to treat certain cancers. Following its discovery as an anti-leishmaniasis agent, miltefosine has been repurposed for managing leishmaniasis, and recent studies have highlighted its antifungal effects on *C. neoformans*. Treatment with miltefosine was shown to increase ROS level in *C. neoformans* H99 cells, like Amp B [47]. In this study, sclareolide showed promising antifungal effects through triggering the oxidative stress-related pathways of *C. neoformans* H99 cells. It is also evidenced by reduced MMP. The amount of oxidative stress developed during sclareolide treatment was not enough the induce cell death. However, this impacted on the cellular components like DNA, proteins, and lipids, contributing to the fungistatic action of sclareolide.

The presence of a carbonyl group in the lactone might facilitate interaction with essential enzymes involved in the cellular metabolism of the organism, contributing to oxidative stress. On the other hand, the decalin motif is found in the structures of many natural products produced by certain microorganisms. Studies also highlighted the biological importance of decalin and its derivatives, which expressed antifungal activity against *C. neoformans* [72,74]. The presence of a bicyclic decalin ring in sclareolide provides structural complexity and facilitates its integration into fungal cell membranes. This integration disturbs the membrane integrity of *C. neoformans*, potentially interfering with nutrient transportation and other essential cellular functions.

Combining Amp B with flucytosine has shown improvement in the survival rate of patients diagnosed with cryptococcal meningitis and is considered as a gold-standard regimen in comparison with mono-therapy using Amp B [75,76]. Moreover, in vitro experiments suggested the use of natural products or synthetic compounds to improve the antifungal property of commercial antifungals against life-threatening fungal infections [77,78,79,80]. The combination of sclareolide with Amp B in this study showed no synergistic effects against fungal cells. However, the observed MIC reduction in Amp B and FICI values are better than previous studies reported. This could help us to develop an alternative solution to overcome fungal infections associated with *C. neoformans* H99.

It is essential to assess the toxicological profile of natural products before progressing to clinical application. Lavae survival was dependent on the concentration of the test compound. To generate an appropriate survival curve, both positive and negative controls were included, showing 0% and 100% survival rates, respectively. Based on the results of the present study, we are recommending 1% Triton X-100 as a positive control to evaluate the toxicity in *G. mellonella* larvae. Furthermore, 100% survival in the negative control confirmed proper handling of the larvae [81]. Melanization is considered a negative indicator of larval viability. In this study, no dark pigmentation or visible melanization was observed in sclareolide-treated larvae, unlike those treated with Triton-X 100. Recently, injection of different concentrations of propolis and copaiba demonstrated non-lethal effects on *G. mellonella* larvae, with an 80% survival rate [55]. Similarly, no lethal effects were observed with geopropolis [82] and *Cinnamomum verum* essential oil [64]. In the present study, sclareolide exhibited no toxicity up to a concentration of 128 µg/mL in *G. mellonella* larvae.

## 5. Conclusions

In the present study, sclareolide demonstrated antifungal activity against *C. neoformans* H99. However, variations in the cell numbers were observed upon exposure to different concentrations of sclareolide. Based on the evidence of various experiments, it is confirmed that sclareolide only controls the growth of *C. neoformans* H99 rather than directly killing the fungal cell. The absence of a caspase marker confirmed that sclareolide does not induce cell death via intrinsic or extrinsic apoptotic pathways. Instead, the antifungal mechanism seems to be through non-lethal cellular stress, possibly involving pathways that cause growth inhibition without triggering programmed cell death. In vivo toxicological experiments proved that a higher concentration of sclareolide was not toxic to the *G. mellonella* larvae tested.

Furthermore, detailed studies to explore molecular pathways involved in cellular respiration and mitochondrial dysfunction should be conducted. These studies might help use to uncover the antifungal potential of sclareolide. On the other hand, optimisation and structural modification of sclareolide could enhance its biological properties against wide range of fungal pathogens as seen in several literatures. While the *G. mellonella* model demonstrated the non-toxic behaviour of sclareolide, the use of mammalian models is encouraged to evaluate its pharmacokinetics in systemic fungal infections. Although initial experiments showed no synergistic effect with Amp B, further screening with other antifungals is recommended to uncover the synergistic effect of sclareolide. This will improve the treatment outcome by reducing the dosage of standard antifungals. Based on these, sclareolide represents a novel antifungal alternative and holds promise for future antifungal drug development.

## Figures and Tables

**Table 1:** Minimal Inhibitory Concentration and effect of the combination between sclareolide and Amp B against reference strain of *Cryptococcus neoformans*.

Compounds	MIC (µg/mL)	FICI	Action
Sclareolide	16	2 to 1	Indifferent effect
Amp B	0.25	NA	NA

FICI—Fractional Inhibitory Concentration Index and NA—Not Applicable.

## Data Availability

The data presented in this study are available on request from the corresponding author.
